# The Effects of Compensatory Auditory Stimulation and High-Definition Transcranial Direct Current Stimulation (HD-tDCS) on Tinnitus Perception – A Randomized Pilot Study

**DOI:** 10.1371/journal.pone.0166208

**Published:** 2016-11-10

**Authors:** Simon Henin, Dovid Fein, Eric Smouha, Lucas C. Parra

**Affiliations:** 1 Department of Biomedical Engineering, City College of New York, 160 Convent Avenue, New York, New York 10027, United States of America; 2 Department of Otolaryngology, Head and Neck Surgery, Mount Sinai Medical Center, New York, NY, United States of America; University of Regensburg, GERMANY

## Abstract

**Background:**

Tinnitus correlates with elevated hearing thresholds and reduced cochlear compression. We hypothesized that reduced peripheral input leads to elevated neuronal gain resulting in the perception of a phantom sound.

**Objective:**

The purpose of this pilot study was to test whether compensating for this peripheral deficit could reduce the tinnitus percept acutely using customized auditory stimulation. To further enhance the effects of auditory stimulation, this intervention was paired with high-definition transcranial direct current stimulation (HD-tDCS).

**Methods:**

A randomized sham-controlled, single blind study was conducted in a clinical setting on adult participants with chronic tinnitus (n = 14). Compensatory auditory stimulation (CAS) and HD-tDCS were administered either individually or in combination in order to access the effects of both interventions on tinnitus perception. CAS consisted of sound exposure typical to daily living (20-minute sound-track of a TV show), which was adapted with compressive gain to compensate for deficits in each subject's individual audiograms. Minimum masking levels and the visual analog scale were used to assess the strength of the tinnitus percept immediately before and after the treatment intervention.

**Results:**

CAS reduced minimum masking levels, and visual analog scale trended towards improvement. Effects of HD-tDCS could not be resolved with the current sample size.

**Conclusions:**

The results of this pilot study suggest that providing tailored auditory stimulation with frequency-specific gain and compression may alleviate tinnitus in a clinical population. Further experimentation with longer interventions is warranted in order to optimize effect sizes.

## Introduction

Tinnitus is the subjective sensation of sound in the absence of actual acoustical stimulation. There is a widespread consensus in the research community that tinnitus originates with some peripheral hearing deficit and that maladaptive central plastic mechanisms subsequently lead to the tinnitus percept [1]. Most tinnitus participants have clear audiometric evidence of hearing loss (e.g. hearing thresholds greater than 20 dB HL or more at some test frequencies). In the few cases where such “clinical” hearing loss is not present, there are nonetheless subtle hearing deficits that can be detected using high-resolution Békésy audiograms, otoacoustic emissions [2,3], or auditory brainstem responses [4]. Our hypothesis is that reduced input to the auditory pathway leads to increased sensitivity (e.g. increased gains in the auditory pathway), which magnifies normal background neuronal activity in the frequency regions of hearing loss, leading to the perception of an actual sound [5]. In this view, auditory stimulation, which compensates for the reduced input (e.g. hearing aids or compensatory stimulation), can potentially revert the maladaptive plasticity. Previously, we have measured tinnitus spectral profiles and compared these to carefully recorded audiograms [3]. We found that they tracked each other fairly well in a subset of participants (2/3 of participants). Thus, we hypothesized that this subset of participants may benefit from auditory stimulation, provided it is carefully tailored to compensate for the subject-specific hearing deficit.

There are also many individuals who have hearing loss but do not present with complaints about tinnitus. One theory stipulates that limbic structures fail to block out aberrant activity that may develop as a result of hearing loss [6]. This is supported by functional imaging data involving limbic structures–an area that has been implicated in emotional responses of aversive stimuli [7]. Anxiety is a common finding among clinical tinnitus patients providing additional support for this explanation [8].

The use of noninvasive neuromodulation techniques to alleviate tinnitus has recently received considerable attention. The thought is that stimulating the brain may help to disrupt or suppress aberrant cortical activity that underlies the tinnitus percept. For example, transcranial magnetic stimulation (TMS) applied to the auditory cortex has been shown to temporarily suppress narrowband and broadband tinnitus [9]. Similarly, treatment with transcranial direct current stimulation (tDCS) in a large cohort of participants showed that stimulation of the dorsolateral prefrontal cortex benefitted 1/3 of participants [10]. More recently, anodal stimulation of the auditory cortex was found to be significantly more effective than cathodal stimulation, suggesting that the disrupting effect of anodal stimulation on neural hyperactivity may provide some benefit to tinnitus suffers [11]. However, there is some doubt as to how long-lasting these effects may be. A recent review of the tDCS literature concluded that most of the studies reviewed reported a temporary reduction in tinnitus loudness or annoyance (14/15), regardless of the exact stimulation parameters and/or location, but none showed any long-term benefit of tDCS [12].

In this study, we combined compensatory auditory stimulation (CAS) with high-definition transcranial direct current stimulation (HD-tDCS) in an attempt to boost re-adaptation of auditory gains to normal levels and to reduce limbic activation. HD-tDCS is a method of non-invasive brain stimulation that uses smaller, ring-type electrodes to deliver more targeted electrical stimulation to specific brain regions than traditional tDCS techniques [13]. The experiment involved exposure to enhanced sound that compensated for subject-specific hearing deficits, while participants watched four episodes of a popular television series to emulate normal sound exposure of daily-living and to distract from the auditory and/or electrical stimulation and thus ensure adequate blinding to the intervention. Concurrent with auditory stimulation, HD-tDCS was administered and tinnitus strength was measured before and after the intervention. Using a randomized Balanced Incomplete Block Design, each subject received CAS and non-compensatory auditory stimulation (AS), paired with either HD-tDCS or sham HD-tDCS.

## Materials and Methods

### Participants

**Fig 1:**
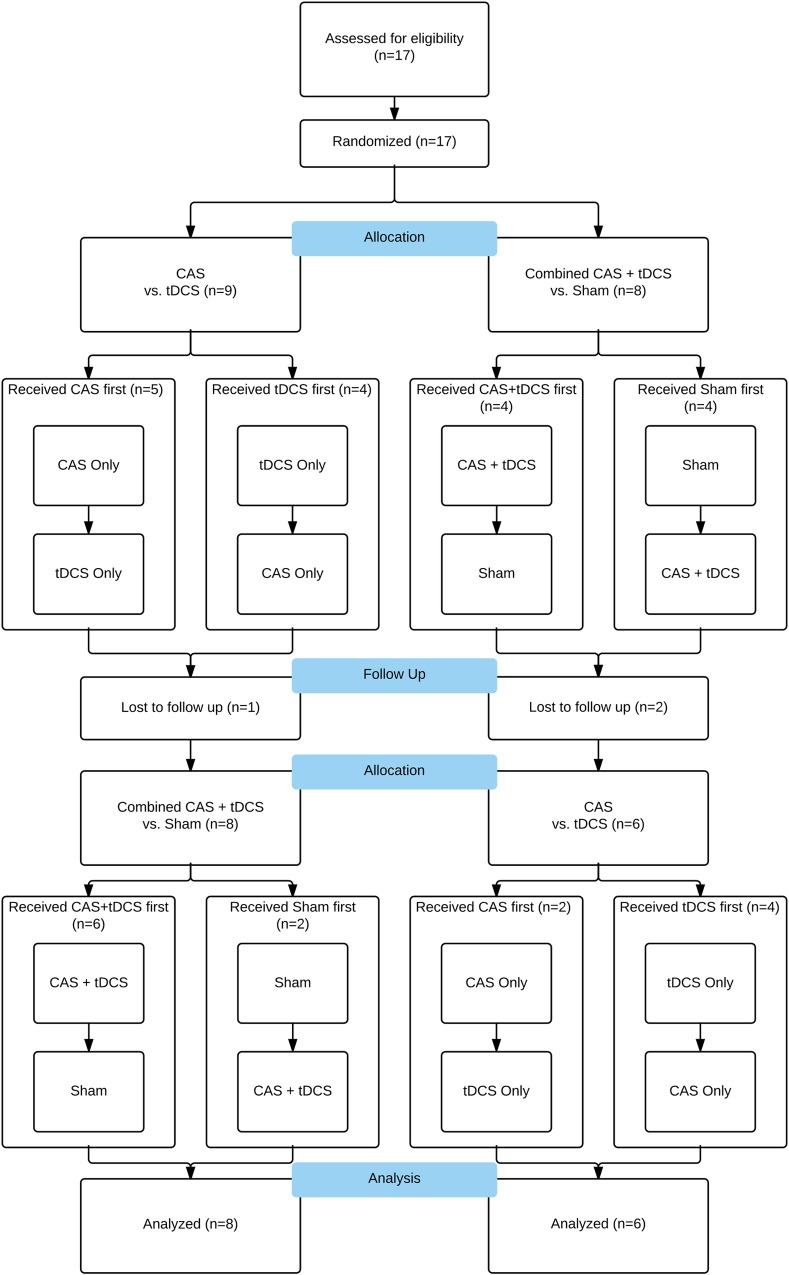
Flow diagram depicting subject allocation, intervention and data analysis. The number of participants completing each portion of the study is indicated within the boxes.

Participants were recruited from the routine neuro-otology clinical patient population at the Mount Sinai School of Medicine. Audiologists recruited suitable patients who reported chronic tinnitus, inviting them to participate in the study. Participants were required to be at least 18 years of age with no more than 50 dB HL hearing loss at any audiometric test frequency, and with no history of chronic skin conditions, cardiac disease, or chronic neurological condition. Based on previously published data on the effects of tDCS on tinnitus rating scales, a preliminary power analysis revealed that at least 14 participants would be required to be able to detect a similar effect with a power of 0.8 (effect size, f = 0.4, see [14]). 17 participants (16 male, 1 female, ranging in age from 28–73 years) participated in the screening session. Of the 17 participants, 14 completed all 3 sessions of the study (1 screening + 2 treatments sessions, see Procedures and Fig 1). Participants that customarily wore hearing aids were asked to take off their aid during all auditory testing and stimulation. Participants completed all test sessions in a sound-attenuating booth. Sony MDR 7506 headphones were used for audiometric testing and for audio playback during video watching. The headphones were calibrated to equalize SPL and provide a flat response across frequencies [cf. 3]. All stimuli and behavioral tasks were presented via custom software developed in MATLAB (The Mathworks, Natick, MA). Recruitment and testing of all participants was performed between April 2013 and July 2014.

### Audiograms

**Fig 2:**
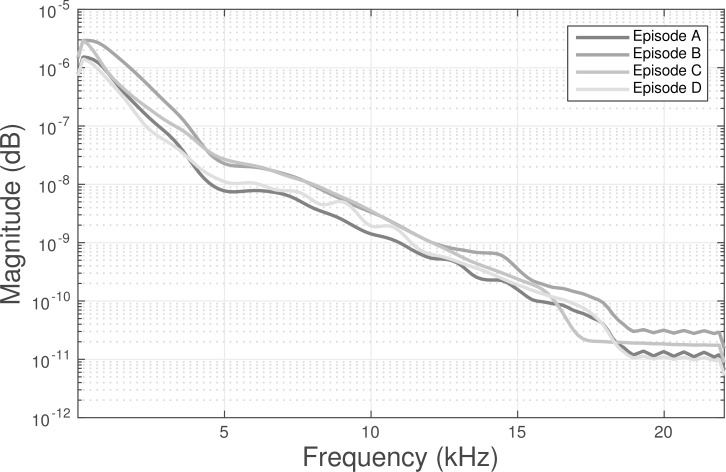
Plot of the mean power spectral density for each of the episodes used in this study.

Audiograms were obtained in both ears using a Békésy tracking procedure at 12 frequency points between 150Hz and 8kHz. Participants were administered the test using a custom computer program. The user interface was relatively simple and asked participants to press and hold a button as soon as they heard a tone (and release it when they could no longer hear it). The audiometric data obtained during the screening session, shown in Fig 2, was used to modify the audio stream of a television program by applying gain to frequencies of moderate hearing loss.

### Minimum masking level and visual analog scales

As the tinnitus percept is subjective, behavioral measures of the tinnitus percept were employed to determine individual tinnitus strength. Behavioral testing included the minimum masking level (MML) test and the visual analog scale (VAS). The MML test consisted of a white noise masker, presented binaurally. The masker was adjusted by the subject via a custom interface, starting at 30dB SPL, in steps of 1 dB, until the tinnitus was no longer perceived, which is defined as the MML (max level 80 dB SPL). At the beginning of the trial, the slider used to adjust the masker level was also positioned all the way to the level (representing 30 dB SPL, a level below each participants MML level) and participants were required to drag the slider to adjust the volume. The VAS consisted of a slider, with which participants recorded their current perceived tinnitus level on a scale from 0–100%, in 1% increments. The slider was positioned at the 50% mark on each repeat of the VAS test.

### Tinnitus likeness spectrum

Using a tinnitus likeness test, a spectral profile of each subject’s tinnitus was ascertained [15]. The test establishes the perceived similarity of the tinnitus percept with test tones at different frequencies. Each subject completed a tinnitus likeness test during each of the three study sessions, and the average of the three tests was used for analysis, including for measuring the consistency in reporting the percept and correlation with audiograms. The test also documented the tinnitus bandwidth (tonal, ringing, hissing), lateralization (left, right, both), and quality (pulsating, steady).

### Auditory compensation

During the intervention we asked participants to watch episodes of a popular television series with the goal of distracting them from the stimulation (auditory/electrical) and assure blinding to the various stimulation conditions. Four episodes from”Family Guy” (Fox Broadcasting Company) were chosen for the treatment sessions. The episode duration was approximately 21 minutes and audio was predominantly human speech (i.e. dialogue). The power spectral density (PSD) was calculated for multiple episodes (N = 23) and we selected the four (4) episodes with the most similar PSDs (Fig 1). The audio soundtrack from two of the four episodes (chosen randomly for each subject) was modified for the compensatory auditory stimulation (CAS) intervention. The audio soundtrack was adjusted by applying a log-linear gain to each of 12 frequency bands such that individual hearing level (HL) thresholds were compensated (Fig 2). With this technique low intensity sounds are amplified more than high intensity sounds. Non-linear compression is implemented so that no gain occurs beyond 70 dB SPL, which is the assumed knee-point above which basilar membrane becomes non-compressive (e.g. linear) [16]. In order to apply these individualized, frequency-dependent gains separately in each of the 12 frequency bands, the sound was decomposed using a Morlet filter bank (center frequencies as in Fig 2 with 1-octave bandwidth), instantaneous amplitudes modulated according to the non-linear gain, and summed across bands to resynthesize the modified sound. The modified audio was then re-spliced with the original video.

### High-definition transcranial direct current stimulation (HD-tDCS)

A custom electrode cap from EASY CAP^®^ was used to hold four custom printed electrode holders, each filled with Sigma conductive gel to deliver high definition transcranial direct current stimulation (HD-tDCS). Electrodes were each used for a maximum of 40 minutes as cathodes and 40 minutes as anodes. A Soterix Medical^®^ 1 x 1 tDCS device and 2 x 2 splitter device were employed to achieve 2 x 2 stimulation. The HD electrodes (annular Ag/AgCl ring-type, outer radius: 12mm, inner radius: 6mm) were placed to target frontal insula and auditory areas bilaterally as both auditory cortex and anterior insula have been implicated in the perception of tinnitus [17], though it is important to note that the level of targeting of deep brain areas is limited. HD-Targets software (Soterix Medical^®^, New York, NY) was used to find the ideal electrode placement for targeting anodal stimulation of the superior temporal gyrus (STG) and nucleus accumbens (Nacc). In this arrangement, cathodal electrodes were placed over lateral prefrontal cortex to suppress limbic activity and anodal electrodes were placed over primary auditory (temporal) areas to boost auditory adaptation. Thus, HD-tDCS stimulation was delivered bilaterally over prefrontal lateral cortex and auditory cortex with opposing polarities. Stimulation consisted of a total of 2mA current split over the four electrodes with each electrode drawing 1mA.

### Procedures

Participants attended an initial screening session, during which they were administered an audiogram, minimum masking level tests (at the beginning and end of the session), and the tinnitus likeness test. In addition, during the initial screening session all participants completed a Beck anxiety inventory [18], Tinnitus Reaction Questionnaire (TRQ) [19], and the Zung self-rated depression scale [20]. Participants all signed an IRB approved consent form before participating in the session.

Episode order and intervention order was randomized for each subject using a balanced incomplete block design (see Fig 1). Randomization was generated by computer program prior to the commencement of the study. Each subject received 4 treatments, split into 2 sessions. CAS and HD-tDCS were set up in a nested crossover design so that each participant received each treatment (CAS-alone, tDCS-alone, CAS+HD-tDCS, and Sham). To minimize the risks to participants, HD-tDCS was only given during one of the 20-minute treatment periods in each of the two sessions. Participants were blinded as to which intervention they were to receive during the duration of the study.

In the CAS condition the sound spectrum was amplified with gains matched to the audiogram measures (as described above). In the non-compensatory condition the sound was unaltered. In all instances sound levels did not exceed what participants are customarily exposed to in daily life (mean of 40 dB SPL and peak levels of no more than 90 dB SPL).

During two of the treatments, participants received 20 minutes of electrical stimulation at 2mA, alone or paired with CAS. For the other two treatments, participants received either ‘sham’ stimulation or received CAS alone (see Fig 1 for allocation details). During the sham sessions, the HD-tDCS device increased current output until reaching 2mA and then immediately reduced the current to 0, where it remained inactive for 20 minutes. At the end of the 20 minutes, the HD-tDCS device again increased current output to 2mA before decreasing to 0mA. Sham stimulation was used to simulate the actual stimulation sessions and maintain the single-blind aspect of the study [21]. No side effects were reported for any of the stimulation conditions used in this study.

### Outcome measures and statistical analyses

Before and after each condition (e.g. CAS, HD-tDCS, CAS + HD-tDCS, Sham) subjects performed the VAS and MML tests. The delay between the end of each intervention (e.g. video) and subsequent behavioral testing was approximately 2 minutes. To assess the effectiveness of each treatment, the difference between pre- and post-tests was computed for each condition (in dB for MML, and percentage points for the VAS). To test our initial hypothesis that CAS combined with HD-tDCS would provide a boost in tinnitus reduction over either treatment alone, we assessed the efficacy of all treatments using a linear mixed-effects model ANOVA with treatment (CAS vs. HD-tDCS) as fixed effects and participant as a random effect. Other factors such as treatment order and video used for treatment could not be included in the model since each participant could not receive every possible order or video + treatment combination to model. However, in the study design, all attempts were made to counterbalance blocks across all subjects and sessions to reduce the effects of order effects within a block (e.g. number of subjects receiving a particular treatment first was not equal across all blocks, refer to Fig 1). In addition, the video used for each treatment was randomized across all individuals and treatments so as to minimize their influence. Model fits were confirmed for normality of residuals and the behavioral results were confirmed for homogeneity of variances across treatments. All statistical analysis was performed using MATLAB (version 2014b, The Mathworks, Natick, Massachusetts).

### Ethical approval

The research protocol was approved by the IRB of the Mount Sinai School of Medicine. The study was not registered before subject enrollment, as it was not a requirement of the Institutional review board. The study has since been registered with ClinicalTrials.org (NCT02648542, January 2016). The authors confirm that all ongoing and related trials for this drug/intervention are registered.

## Results

We recruited a total of 17 participants from among the patients presenting with tinnitus at the Mount Sinai School of Medicine. Of these, 14 completed the screening and 2 treatment sessions, which, on average, took approximately 3 weeks to complete. Table 1 shows the baseline demographic data for the group of participants that completed the study.

**Table 1:** Baseline demographic and tinnitus related data for the group of participants that completed the study.

**Gender**	10 M, 4 F
**Mean age in years (SD)**	55.2 (6.8)
**Depression (Zung depression scale)**	26.7 (8.6)
**Anxiety (Beck Anxiety Inventory)**	8.8 (8.3)
**Tinnitus Reaction Questionnaire**	18.4 (16.4)
**Baseline VAS (0–100)**	66.5 (23)
**Baseline MML (dB SPL)**	63.4 (13.9)

Compensatory auditory stimulation (CAS) was customized for each subject by compensating for frequency-dependent hearing losses based on their respective audiograms (see Fig 2).

**Fig 3:**
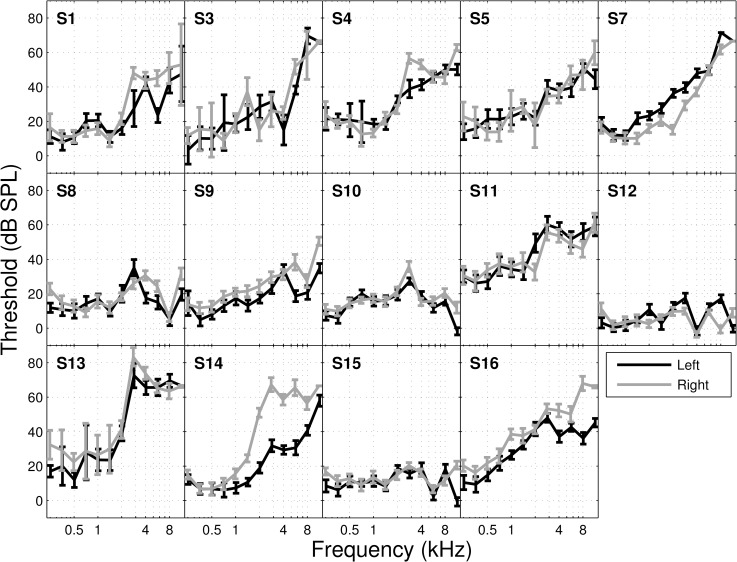
Audiograms for all participants who completed the study. Audiograms for each subject are represented in dB SPL from 250 Hz up to 11.3 kHz (2 points per octave). Left and right ears are shown as black and grey lines, respectively.

Qualitative descriptors of the tinnitus percept for the 14 participants are reported in Table 2. 50% of participants (7) provided consistent responses on all three descriptors (location, bandwidth and periodicity) during each of the three sessions. In this cohort tinnitus likeness (TL) ratings (Fig 3) generally followed the audiograms, which is consistent with previous studies [e.g. 3,15]. When comparing across all three TL measurements obtained, only 36% (5) of participants showed reproducible results (e.g. significant correlation, pearson’s R, between likeness ratings obtained in different sessions at alpha level < 0.05).

**Table 2:** Summary of qualitative data from all participants.

Subject ID	"What does it sound like?" (Tonal/Ringing/Hissing)	"Is it steady or pulsing?"	"On which side is your tinnitus?" (Left/Right/Both/Unsure)	TRQ Score
1	Hissing	Steady	Left	41
3	INC	Steady	Both	25
4	Hissing	Steady	Both	23
5	Hissing	Steady	Left	3
7	Hissing	Steady	INC	1
8	INC	INC	Left	9
9	Hissing	Steady	Both	8
10	INC	INC	INC	46
11	INC	Steady	Both	6
12	Tonal	Steady	Both	2
13	Tonal	Steady	Left	48
14	Hissing	Steady	Left	9
15	Tonal	Steady	INC	13
16	Hissing	Steady	INC	23
% Consistent	71.43%	85.71%	71.43%	NA

Qualitative data describing tinnitus percept, as recorded from 3 tinnitus likeness tests. (INC: inconsistent responses recorded for a particular question across all 3 tests).

Table 2 also provides TRQ scores for each subject. Out of a possible maximum score of 104 on the TRQ, participants reported in the range of 1 to 48 with a median of 11, indicating that this cohort had only mild tinnitus [19].

Changes in severity of tinnitus were measured immediately before and after the 4 interventions using minimum masking level (MML) and visual analog scale (VAS) tests (Fig 5, see also S1 Table.). Two subjects (Subject ID 14 and 15) were identified as outliers on the VAS outcome measure, performing at ceiling on this test (e.g. VAS Score = 100 out of 100 on pre/post treatment testing during 2/4 treatments) and were therefore removed from subsequent analysis on the VAS measure. Generally, both measures trended lower with treatment, suggesting a possible improvement. A mixed-effects model ANOVA was performed (Table 3) and revealed a significant decrease in the MML for the CAS treatment (F = 6.822, p = 0.022) and a trend towards significance on the VAS metric for CAS (F = 4.377, p = 0.06). There was a numerical improvement on both measures with HD-tDCS (see Fig 5) but effects were not significant. Not surprisingly, significant interactions were observed on the MML test for each treatment and participant, indicating that treatment effects varied across participants.

**Table 3:** ANOVA table for MML and VAS tests.

	***MML***				
**Source**	**SS**	**d.f.**	**MS**	**F**	**P**
**CAS**	50.161	1	50.161	6.822	**0.022**
**HD-tDCS**	6.446	1	6.446	0.565	0.466
**Participant**	138.304	13	10.639	0.649	0.785
**CAS x HD-tDCS**	0.018	1	0.018	0.008	0.932
**CAS x Participant**	95.589	13	7.353	3.110	**0.025**
**HD-tDCS x Participant**	148.304	13	11.408	4.826	**0.004**
	***VAS***				
	**SS**	**d.f.**	**MS**	**F**	**P**
**CAS**	165.021	1	165.021	4.377	0.060
**HD-tDCS**	180.188	1	180.188	3.246	0.099
**Participant**	1713.229	11	155.748	2.228	0.103
**CAS x HD-tDCS**	2.521	1	2.521	0.108	0.748
**CAS x Participant**	414.729	11	37.703	1.619	0.219
**HD-tDCS x Participant**	610.563	11	55.506	2.383	0.083

Constrained (Type III) sum of squares. Significant results are shown in bold. (CAS: Compensatory auditory stimulation, HD-tDCS: High-Definition Transcranial Direct Current Stimulation)

## Discussion

The primary aim of this study was to prospectively assess whether CAS, HD-tDCS, or both can provide significant relief of tinnitus. We hypothesized that the strength of the tinnitus percept is related to the specific hearing loss, and thus predicted that tinnitus could be reduced by providing compensatory auditory stimulation matched to an individual’s frequency-specific hearing loss. In addition, we hypothesized that HD-tDCS would promote re-adaptation of cortical structures and help reduce the tinnitus percept.

In this small cohort of clinical tinnitus participants, we found that CAS did indeed reduce the strength of the tinnitus percept as measured using minimum masking level, and trended towards significance with the visual analog scale. In addition, as seen in Fig 5 (bottom panel), VAS reductions found for CAS and HD-tDCS individually seem to produce a synergistic effect when combined (e.g. HD-tDCS+CAS > HD-tDCS/CAS alone). However, the large variability observed under the sham treatment precluded these treatment effects from being significant in this small sample of participants. While the VAS measure has previously been shown to be reliable [22], it may require considerable practice in order to achieve high accuracy [23]. In this study, participants were not provided extensive practice with the scale and therefore the lack of a significant reduction in the VAS is not entirely unexpected. Some of this variability is also explained by the adaptation level theory [24], which posits that tinnitus magnitude (loudness, annoyance) can vary depending on the context and a subject’s emotional state. In contrast, more objective psychoacoustic measures, such as the MML, are more robust to these confounds and may provide a more meaningful estimate of tinnitus magnitude.

**Fig 4:**
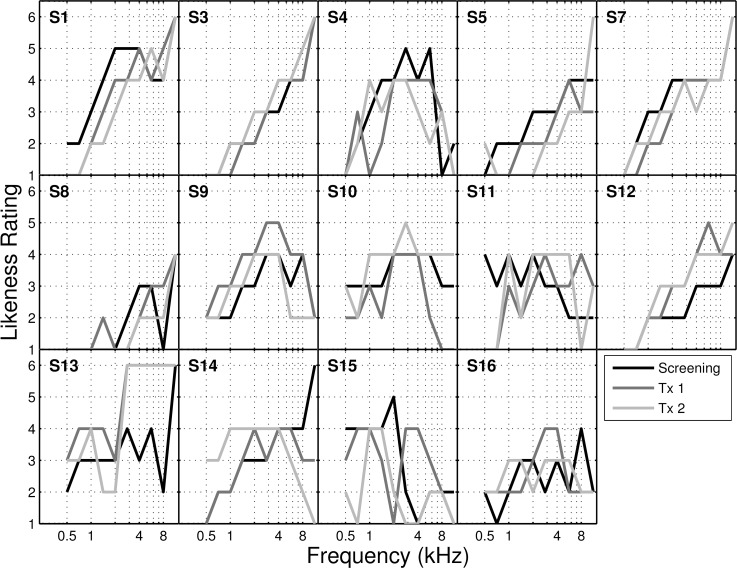
Tinnitus likeness spectrum. Tinnitus likeness rating provided by all participants at initial screening (Screening) and before each treatment session (Tx 1 & Tx 2).

While the relatively small reduction in subjective tinnitus observed in this study (i.e. approximately 2.4 dB and 3.43% for CAS alone for MML and VAS, respectively, see Fig 4) are similar to those previously reported for acute interventions [25], the fact that larger effects were not observed may in part be due to specific subject population used in this study. Participants exhibited only modest TRQ scores (median = 11 and range = 47, on TRQ scale). As these instruments have been thoroughly validated in the literature [23], this potentially indicates that this subject population is not entirely representative of tinnitus sufferers on the whole. Nonetheless, CAS still provided a temporary reduction of the tinnitus percept for this set of participants.

**Fig 5:**
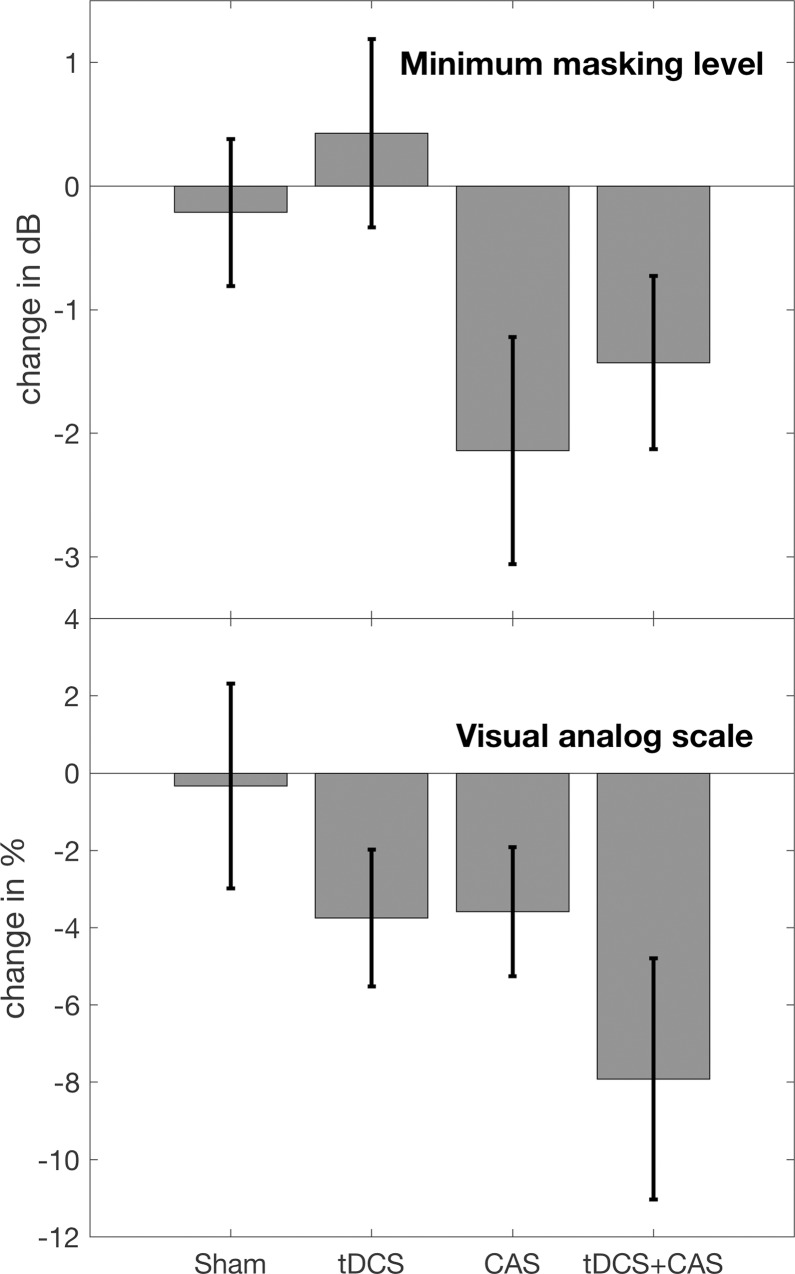
Change in MML and VAS with treatment. Change in minimum masking levels and visual analog scale tests under the four treatment conditions (MML: N = 14, VAS: N = 12). Sham refers to non-compensated AS along with sham HD-tDCS to which participants were blinded (see Procedures for details). Error bars represent the standard error of the mean (SEM).

Tinnitus suffers often report a temporary reduction in their tinnitus percept immediately following masking sound, termed residual inhibition (RI)[26]. While RI cannot be completely ruled out due to the relatively short interval between the end of stimulation and behavioral testing (e.g. approx. 2 minutes), we believe RI cannot be a major contributing factor for two reasons: (1) the effects of RI typically only last for less than one minute [27], and (2) we do not see reductions in VAS or MML scores in the sham condition even though this condition consisted of un-compensated auditory stimulation, which on its own would have also induced RI.

Contrary to our prediction, but in line with previously published results [25,28], we did not find a significant effect of HD-tDCS on reducing the tinnitus percept. However, in this study electrode position differed slightly from earlier work in that 2mA were split across two sets of electrodes, and were presumed to target slightly different cortical areas. In addition, an effort was made to enhance blinding by distracting participants during stimulus presentation by presenting videos. One potential limitation of the current study design was that two different treatments were included in each session. While care was taken not to include two treatments of HD-tDCS or CAS within one session, this may not have allowed for enough ‘washout’ between treatments within one session, obscuring some of the potential benefits of HD-tDCS. However, this methodological thread seems tenuous as a definitive improvement was observed using CAS alone, indicating that at worst, a similar improvement would have been observed with HD-tDCS alone. Due to time constraints imposed to maximize subject retention over the duration of the study, each participant was not exposed to every possible treatment order, nor were they exposed to every combination of video + treatment. However, we believe that the block design structure used, along with the nested cross-over design of the treatment blocks within subjects, and randomization of videos across treatments and subjects, helped to minimize any possible influence of these factors. Future studies might consider using different treatments arms for each treatment, and fixing the stimulus material to rule out confounding factors. Nonetheless, more research is required to assess if the boosts in neural plasticity that we hypothesized would occur with HD-tDCS would also be observed with additional treatments and over a longer course of intervention. Similarly, more research is needed to assess whether the improvements observed using CAS would remain after several days or even weeks.

## Conclusions

When tailored to match a subject's frequency-specific hearing loss, brief CAS intervention provides a short-term reduction in the tinnitus percept, suggesting that long-term intervention with CAS may be promising. As this auditory stimulation works using natural stimuli, its long-term effects could be assessed using hearing aids and any positive results would immediately translate to clinical practice. While transcranial direct current stimulation has previously been shown to provide temporary reductions in the tinnitus percept, this was not observed in the present study. However, further research is necessary to assess whether repetitive treatments of HD-tDCS may be effective over longer periods, especially when paired with compensatory auditory stimulation.

## Supporting Information

S1 File CONSORT Checklist. (DOC)

S2 File Study Protocol. (DOC)

S1 Table Summary Results Table. For each subject, table shows treatment order, change in behavioral measures (change from baseline, post-pre), and episode number used in each treatment.
(DOCX)
