# Supplementary material for: The Effects of Compensatory Auditory Stimulation and High-Definition Transcranial Direct Current Stimulation (HD-tDCS) on Tinnitus Perception – A Randomized Pilot Study
Source: PLoS One. 2016 Nov 10;11(11):e0166208. doi: 10.1371/journal.pone.0166208 (PMC5104367; doi:10.1371/journal.pone.0166208)
Supplement: S2 File — (DOC) [file pone.0166208.s002.doc]

# Study Protocol

**Brief Summary of Research (250-400 words):**

Tinnitus refers to persistent “ringing in the ears” in absence of external sound. For some individuals tinnitus can be disabling. Most tinnitus is of idiopathic origin and currently there are no reliable treatment options available. Existing approaches, such as hearing aids, counseling, and noise masking typically provide only partial relief and only for a minority of patients. Newer exploratory methods include tailored auditory stimulation and various brain stimulation techniques. While these techniques show great promise in a subset of subjects (maybe about 1/3), it is hard to anticipate which, if any, will benefit a given patient.

Of the electric stimulation techniques, transcranial Direct Current Stimulation (tDCS) has the lowest risk profile. It applies small currents to the scalp non-invasively for short periods of time (2mA, 20 min). Preliminary studies have shown benefit in 30% of patients, with significant relief after stimulation (30% reduction in tinnitus intensity and distress). Side effects include a slight tingling sensation at the beginning of simulation. Sometimes there is a light skin irritation at the site of stimulation, which resolves spontaneously after a few hours. tDCS has been explored for a number of neurological conditions, and it is most effective when paired with some form of training (e.g. motor rehab after stroke). For tinnitus however this treatment modality has only been tested in isolation. The purpose of this pilot study is to explore the benefits of combining tailored compensatory auditory stimulation with tDCS.

There is widespread consensus in the research community that tinnitus originates with some peripheral hearing deficit, and that maladaptive central plastic mechanisms subsequently lead to the tinnitus percept. Most individuals with tinnitus have audiometric evidence of hearing loss. Our hypothesis is that reduced input to the auditory pathway leads to increased sensitivity (auditory gain) and finally aberrant activity in the frequency band with hearing loss. In this view, auditory stimulation that compensates for the reduced input can potentially revert the maladaptive plasticity. This is consistent with the reported benefits of hearing aids and tailored auditory stimulation. Our hypothesis is that tDCS can boost adaptation induced by compensatory auditory stimulation to reduce the strength of the phantom percept.

1. Objectives

Our objective is to reduce the severity of tinnitus for patients. We will attempt this on patients in whom the tinnitus percept correlates with their specific hearing loss profile. To this end we will apply compensatory auditory stimulation (CAS) delivering stronger stimulation in the frequency bands of hearing loss and combine this with tDCS to facilitate adaptation. We will test 10 tinnitus patients in two separate sessions combining the two stimulation techniques in a 2x2 balanced crossover design (tDCS and sham-tDCS, CAS and non-CAS). Each treatment is delivered over 20 minutes and changes in tinnitus are measured before and after these acute interventions. Patients are selected from Dr Smouha’s clinical practice of otology - first based on their audiograms and tinnitus assessments, as well as ratings on various normed questionnaires. We anticipate that 15 but no more than 20 patients will participate in the first selection session. The 10 selected subjects participate in a total of 3 sessions (1 selection, 2 treatment). The goal of this small preliminary trial is to establish feasibility. If successful, the data will provide the basis for a NIH RO1 application to support a full clinical trial.

1. Background

Various treatment protocols have been proposed for tinnitus including hearing aids, auditory masking, psychological counseling, tailored auditory stimulation, and various brain stimulation techniques (transcranial Direct Current Stimulation – tDCS, Transcranial Magnetic Stimulation – TMS, vagus nerve stimulation – VNS, Deep-Brain Stimulation – DBS). Many of these techniques show promise on a subset of subjects but it is hard to anticipate which will benefit what type of tinnitus patient. Of the electric stimulation techniques, tDCS has the lowest risk profile. It applies small currents of no more than 2mA. At typical contact resistances this amounts to no more than 10V (and in all instances less than 30V, which is below the safety limit for exposed wires in home appliances, e.g. halogen light fixtures). The purpose of this pilot study is to explore the benefits of combining tailored auditory stimulation with tDCS.

There is a widespread consensus in the research community that tinnitus originates with some peripheral hearing deficit and that maladaptive central plastic mechanisms subsequently lead to the tinnitus percept. Most tinnitus subjects have clear audiometric evidence of hearing loss (with hearing thresholds increased by 20dB or more at some test frequencies). In the few cases that present without such “clinical” hearing loss, there are nonetheless subtle hearing deficits which can be detected using Bekesy audiograms, otoacoustic emissions (Zhou 2010), or auditory brain stem responses (Schaette 2011). Our hypothesis is that reduced input to the auditory pathway leads to increased sensitivity (auditory gains) and finally aberrant activity in the loss bands (Parra, 2007). In this view, auditory stimulation which compensates for the reduced input, e.g. hearing aids or compensatory stimulation like in the FDA approved treatment of Neuromonics, can potentially revert the maladaptive plasticity. We have measured tinnitus spectral profile and compared this to carefully recorded audiograms (Zhou 2010). We find that they track each other fairly well for a subset of subjects (2/3 of subjects). We predict that this set of subjects are the most likely candidates to benefit from auditory stimulation, provided it is carefully tailored to compensate for the subject-specific hearing deficit.

One additional aspect merits consideration. There are many subjects that have hearing loss but do not develop tinnitus. One theory stipulates that limbic structures fail to block out aberrant activity that may develop as a result of hearing loss (Rauschecker 2010). This is supported by some functional imaging data involving limbic structures – an area that has been implicated in emotional responses of aversive stimuli (Leaver 2011). A high level of anxiety is a common finding in clinical tinnitus patients. Treatment with tDCS on a large cohort of subjects Has shown that dorsolateral prefrontal cortex stimulation benefits 1/3 of subjects (Vanneste 2010). Our modeling studies on current flows suggest that this form of stimulation may indeed have reached limbic areas (unpublished, based on Dmochowski 2011). In general tDCS is used in combination with a behavioral and perceptual training paradigm and stimulation acts to boost training effects. However, the tDCS study on tinnitus performed electric stimulation alone (Vanneste 2010). A study using vagus-nerve stimulation (invasive) combines electric with auditory stimulation and has shown promising results in animal studies (Engineer 2011). The premise of that study is that central adaptation, induced by auditory stimulation, is facilitated by vagus-nerve stimulation. The approach taken here parallels this technique but using non-invasive transcutaneous electric stimulation instead.

Specifically, we would like to combine compensatory auditory stimulation with tDCS to boost re-adaptation of auditory gains and to reduce limbic activation. We propose a pilot study with 10 tinnitus subjects carefully selected based on audiogram and tinnitus spectrum. We will exclude subjects for whom there is no clear correspondence between audiogram and tinnitus profile as measured by the “tinnitus likeness spectrum” (Zhou, 2010). We will also exclude subjects that show depression or anxiety using normed questionnaires as we believe that these subjects are less likely to benefit from auditory stimulation. The experiment involves watching a feature-length movies at comfortable listening levels with sound enhancement, typically at high frequencies, to compensate for the participants’ specific hearing deficits. Concurrent with auditory stimulation tDCS will be administered with standard parameters (2mA, 20 min). Before and after the stimulation the participant will be asked to assess the strength of his/her tinnitus. While we are interested in the compensatory auditory stimulation (CAS), the movie is intended to avoid focusing attention to the auditory percept, which is typically counterproductive. Each subject will receive CAS and non-compensatory AS and this will be paired with tDCS or sham-tDCS. Each intervention takes 20 minutes + 10 minutes for tinnitus assessment and an initial assessment at the beginning. To test all 4 conditions of the 2x2 crossover design would thus take over two hours. Hence we intend to divide the experiment into two sessions, each lasting no longer than 1.5h. An additional session is required for preliminary testing where we will obtain high-resolution audiograms and Tinnitus Likeness Spectrum. In total each patient will participate in 3 sessions.

1. Setting of the Human Research

The PI of this project is a neuro-otologist at MSSM, Dr. Eric Smouha, who has many years of clinical experience with tinnitus patients. The co-PI and investigator are a neurosurgeon Dr. Brian Kopell of MSSM who has experience with deep-brain stimulation in a cohort of tinnitus subjects and Prof Lucas Parra of CCNY. He has experience in tinnitus assessment and extensive experience with tDCS. He is the PI for a current clinical trial on HD-tDCS for aphasia rehabilitation after stroke and the PI for a study on the basic mechanisms of DC and AC transcranial stimulation. The audiologist Karen Siegel who works with Eric Smouha will assist in recruitment and audometric testing. All experiments will be performed at MSSM in the offices of Dr. Eric Smouha at Mount Sinai Medical Center. Audio test booths are available there with the required acoustic shielding. Equipment for audiometric testing and audiovisual stimulation will be provided by Dr. Parra. tDCS equipment will be provided at no charge by Soterix Medical Inc.

1. Resources Available to Conduct the Human Research

The audiogram and tinnitus assessment tools have been developed by Dr. Parra. They run on a laptop computer and use calibrated headphones which will be provided for this study at no cost. The experiments will be conducted by Dr. Parra and his students in collaboration with Drs. Kopell and Smouha. A CCNY startup company, Soterix Medical Inc, will provide the tDCS equipment and supplies at no cost. Electrode placement requires computational modeling, an expertise that is available at the lab of Prof Parra.

1. Study Design
   1. Recruitment Methods

Subjects will be recruited from the routine clinical patient population of Dr. Eric Smouha. He or his audiologist will directly approach suitable patients inviting them to participate in the study.

Subjects will be asked to volunteer for theses studies and will receive no compensation. A session may last up to 1.5 hours and each subject may participate in at least one, but no more than 3, sessions.

- 1. Inclusion and Exclusion Criteria

When inviting individuals to participate and prior to scheduling their participation and prior to consenting, subjects will be presented the list of exclusion criteria (see attachment). To avoid privacy concerns and to assure truthful response to these exclusion criteria we will not ask subject specific questions about their physical or mental conditions. Instead we will ask subjects to carefully review these exclusion criteria and ask them to consider if they would like to participate:

Subjects will be verbally re-advised on the nature of the study and on the exclusion criteria before each experiment. For colleagues it is indicated in the consent form and verbally that their participation is completely voluntary and can be discontinued at any time. Their inability or lack of interest in participating will not be questioned and will not affect the work relationship in any way.

In the first screening session subjects will be selected for treatment if they provide reliable tinnitus likeness spectra (correlation coefficient of two separate measures higher than 0.5, r>0.5) and if these spectra correlate with their audiogram (again, r>0.5). If subjects are selected they will be asked to participate in the two treatment sessions.

A standard assessment protocol will be used to determine the severity of tinnitus (Tinnitus Functional Index, Meikle 2011). The TFI is a normed assessment tool that has been developed by an international panel of experts and is recommended for evaluating treatment outcomes in research as well as in the clinic. All persons between 18-80 years of age are eligible to participate in this study. However, subjects with otological disease will be excluded. Subjects with hearing loss, hyperacusis and/or tinnitus are eligible to participate in this study. However, hearing loss should be limited to no more than 50dB so as to ensure effective auditory stimulation in the loss bands. We will exclude subject that show depression or anxiety. We will adopt the Zung self-rating Depression Scale and exclude subjects with a rating above 50. We will also use the Beck Anxiety Inventory and exclude subjects with rating above 36. Both inventories are attached.

Persons of under-represented group and women will not be discriminated against and may take part in this experiment. However, children (<18 years of age) are excluded from this study, as pediatric tinnitus is not the focus of the current project.

- 1. Number of Subjects

We will recruit 10 tinnitus subjects that fit our selection criterion. This implies that the first screening session may include a larger number of subjects, we estimate 15 but hopefully no more than 20.

- 1. Study Timelines

We anticipate accruing subjects at a rate of no less than 2 per month. We hope to complete the study in 6 months and no more than 12 months.

- 1. Study Endpoints

Tinnitus likeness ratings, tinnitus intensity and distress on a visual analog scale (VAS), tinnitus minimum masking levels (MML). All will be measured immediately before and after each intervention.

- 1. Procedures Involved in the Human Research

In the first screening session we will obtain audiograms and tinnitus likeness spectrum following Zhou et al, 2011 (see brief description below). Subjects will also complete the three questionnaires listed above (Tinnitus Functional Index, Zung self-rating Depression Scale, and Beck Anxiety Inventory). Subjects will then be randomly assigned, in a single-blind fashion, to one of two treatment arms. In the treatment sessions we will administer auditory stimulation (compensatory or non-compensatory) and tDCS stimulation (active or sham).

Audiograms: Audiograms will be obtained using Bekesy tracking at 12 frequency points for left and right ear. For this subjects will use calibrated headphones and a custom computer program that runs on a laptop computer. The user interface is relatively simple and asks subjects to press and hold a button as soon as they hear a tone (and release it when they no longer hear it). We can obtain these audiograms in about 30 minutes time.

Tinnitus Likeness Spectrum: Using the same headphones and computer we obtain a rating from the subjects as to how much a given tone sounds like their tinnitus. This is a well established procedure and is fairly easy for subjects to complete in about 10-15 minutes. This test will be performed twice – at the beginning and end of the first evaluation session.

Auditory Stimulation: Subjects will watch a movie using headphones with sound levels adjusted by the subject for comfort. In the compensatory stimulation condition the sound spectrum will be amplified with gains matched to the audiogram measures. In the non-compensatory condition sound will be unaltered. Each session will last no more than 20 minutes.

tDCS: After the start of audio/video display we will commence transcranial electric stimulation, which will last for 20 minutes. We will use “high-density” electrode montages (HD-tDCS). This technique uses several small electrodes to precisely control the focus of stimulation in the brain. These stimulating electrodes use gel and are held in place the same way as EEG electrodes (Signa Gel and EasyCap). HD electrodes will be placed aiming to target left frontal insula and auditory areas, though one should keep in mind that the level of targeting of deep brain areas is limited. Cathodal electrodes (negative polarity) will be placed over lateral prefrontal cortex to suppress limbic activity. Anodal electrodes (positive polarity) will be placed over auditory (temporal) areas to boost auditory adaptation. Thus tDCS stimulation would be delivered over prefrontal lateral cortex and auditory cortex with opposing polarities. Stimulation will use a total of 2mA current split over multiple electrodes (no less than 4 and more more than 8 electrodes) with each electrode drawing at most 1mA. Following common practice, in the sham tDCS condition we will place the electrodes in the same fashion but stimulation will be turned on only at the beginning and end of the 20 minutes period. In the two treatment sessions, separated by one week, tDCS will be active only during one 20 minute period.

Before and after the experiment the participant will be asked about the intensity of his/her tinnitus using a visual analog scale as well as distress. We will also measure minimum masking levels, i.e. volume of a white-noise masker is varied until the tinnitus percept is not longer audible.

- 1. Specimen Banking

N/A

- 1. Data Management and Confidentiality

The data will be saved and identified with an assigned number along with the time of the recording. We will note subjects’ gender and age. Subjects name will not be recorded except on the consent form. A list associating subject number with name will be kept locked and secured (password protected) at the offices of the co-PI Eric Smouha where the experiments will take place. The PI and research assistants from CCNY will only have accessed to the de-identified data. However, the PI may inspect the full records at MSSM to confirm integrity and consistency of the datasets that are recorded over several sessions. The results of these experiments may be used for publication but will not include subjects’ name.

- 1. Provisions to Monitor the Data to Ensure the Safety of subjects

We will monitor outcomes on the tinnitus loudness tests (VAS and MML) right after the intervention. If we find a consistent increase in tinnitus in any of the treatment conditions we will interrupt the study. We will also provide an exit interview at the end of each treatment session to probe for potential negative effects of the stimulation (although none of these and anticipated based on previous literature). We will also follow-up with a call to the subjects on the subsequent day and inquire if their tinnitus has gotten better, worse, or is unchanged or if they had any unusual reactions to the stimulation (see scripted phone dialog).

The following criteria will be used for stopping the study and for reporting unanticipated adverse events to the IRB:

- Any increase in tinnitus intensity of more than 3 points on the VAS with a range of 1-7.
- Any increase in minimum masking level of more than 20dB.
- Any effects queried in the exit interview (pain, headache, fatigue, mood changes etc.) judged by the subject to be severe (3 or 4).
- Any other serious and unexpected adverse event that appears to be an effect of exposure to tDCS reported on the follow-up interview.
  1. Withdrawal of Subjects

Subject may withdraw at any time without repercussion to their clinical treatment.

1. Risks to Subjects

There are no risks factors associated with the present auditory stimulation. However, it is known that when tinnitus patients pay particular attention to faint sounds it may make their tinnitus more noticeable. The viewing of a movie may help to mitigate this risk factor. This attentional modulation should subside after the experiment. A prolonged attentional effect may be more prevalent in anxious subjects. We therefore exclude those subject from this study.

The basic comfort and safety of the gel-based HD-tDCS electrodes has been previously established by the neural engineering lab at CCNY (Minhas 2010). In general, tDCS is associated with tingling sensations in particular at the beginning of stimulation. Sensation is also minimized by ensuring that contact resistance is low before the start of stimulation (40 kOhm or less). At the maximum intensity of 1mA at any one electrode this means a voltage of no more than 40V. It should be noted that resistance typically drops to no more than 10kOhm after a few seconds of stimulation. To minimize sensation during this initial time, currents are usually ramped up slowly typically in a period of 30s. Slight reddening of the skin at the site of stimulation is also possible in some individuals, but this resolves mostly within a few hours or a day at most. All other side-effects (headache, nausea, etc) are infrequent and occur no more often than in placebo treatment (Iyer 2005, Poreisz 2007).

See item i) above for a plan on how we will monitor for any of these effects.

1. Provisions for Research Related Injury

Because the risk profile of tDCS is very low, no research-related injury is anticipated. In the unlikely event that a patient reports any harm from the study, he will be immediately evaluated by Dr. Smouha or his staff, and receive the appropriate medical intervention.

1. Potential Benefits to Subjects

Subjects should not expect direct benefits in this study as we have no evidence that the proposed treatment will be beneficial. However, it is our aim that the strength of the tinnitus percept is reduce at least for a short period of time after the CAS and tDCS sessions.

We will provide patients also with a copy of their high-resolution audiograms, which some patients may find useful.

Subjects will not receive payment for their participation.

There is an indirect benefit associated with the possibility that this study is successful and that it may lead in the future to an effective treatment protocol for tinnitus. However, we will be clear to explain the investigational nature of this study to the participants.

1. Provisions to Protect the Privacy Interests of Subjects

See Data Management plan above.

1. Economic Impact on Subjects

none.

1. Payment to Subjects

none.

1. Consent Process

When subjects are invited to participate in the study, Dr. Smouha will provide them with a copy of the consent form and highlight the list of exclusion criteria. Once the subject comes to the first screening session, the PI or one of the co-investigators will review the consent form with the subject and explain verbally the purpose of the study, the procedures, as well as risks and benefits.

1. Process to Document Consent in Writing

After this review of the consent form and prior to the start of the first session the PI or one of the co-investigators will obtain written consent with a signature of the patient on the consent form.

1. Vulnerable Populations

N/A

1. Multi-Site Human Research (Coordinating Center)

N/A

1. Community-Based Participatory Research

N/A

1. Sharing of Results with Subjects

We will provide a copy of the audiograms.

1. IRB Review History

N/A

1. Control of Drugs, Biologics, or Devices

*Note: The IDS has its own forms that must be completed and a review process that must be followed before the IDS representative will sign off on Appendix B for submission to the PPHS.*

HD-TDCS is a minimum risk procedure and the FDA has designated that the device does not require an IDE (investigation device exception). See attached letter from the FDA.

**References**

Dmochowski JP, Datta A, Bikson M, Su Y, Parra LC, “Optimally focal noninvasive electrical stimulation,” Neural Engineering, 8(4): August 2011.

Engineer ND, Riley JR, Seale JD, Vrana WA, Shetake JA, Sudanagunta SP, Borland MS, Kilgard MP. Reversing pathological neural activity using targeted plasticity. Nature. 2011 Feb 3;470(7332):101-4.

Iyer MB, Mattu U, Grafman J, Lomarev M, Sato S, Wassermann EM. Safety and cognitive effect of frontal DC brain polarization in healthy individuals. Neurology. 2005 Mar 8;64(5):872-5.

Leaver AM, Renier L, Chevillet MA, Morgan S, Kim HJ, Rauschecker JP. Dysregulation of limbic and auditory networks in tinnitus. Neuron. 2011 Jan 13;69(1):33-43.

Meikle MB, Henry JA, Griest SE, Stewart BJ, Abrams HB, McArdle R, Myers PJ, Newman CW, Sandridge S, Turk DC, Folmer RL, Frederick EJ, House JW, Jacobson GP, Kinney SE, Martin WH, Nagler SM, Reich GE, Searchfield G, Sweetow R, Vernon JA. The tinnitus functional index: development of a new clinical measure for chronic, intrusive tinnitus. Ear Hear. 2012 Mar-Apr;32(2):153-76.

Minhas P, Bansal V, Patel J, Ho JS, Diaz J, Datta A, Bikson M. Electrodes for high-definition transcutaneous DC stimulation for applications in drug delivery and electrotherapy, including tDCS. J Neurosci Methods. 2010 Jul 15;190(2):188-97.

Parra LC, Pearlmutter BA, “Illusory percepts from auditory adaptation”, Journal of the Acoustical Society of America, 212(3), 1632-1641, 2007.

Poreisz C, Boros K, Antal A and Paulus W. Safety aspects of transcranial direct current stimulation concerning healthy subjects and patients. Brain Research Bulletin 2007;72:208-214

Rauschecker JP, Leaver AM, Mühlau M. Tuning out the noise: limbic-auditory interactions in tinnitus. Neuron. 2010 Jun 24;66(6):819-26.

Vanneste S, Plazier M, Ost J, van der Loo E, Van de Heyning P, De Ridder D. Bilateral dorsolateral prefrontal cortex modulation for tinnitus by transcranial direct current stimulation: a preliminary clinical study. Exp Brain Res. 2010 May;202(4):779-85.

Zhou X, Henin S, Long G, Parra LC, “Impaired Cochlear Function Correlates with the Presence of Tinnitus and Its Estimated Spectral Profile”, Hearing Research, 277(1-2): 107-116, July 2011.
