# Supplementary material for: The Effects of Compensatory Auditory Stimulation and High-Definition Transcranial Direct Current Stimulation (HD-tDCS) on Tinnitus Perception – A Randomized Pilot Study
Source: PLoS One. 2016 Nov 10;11(11):e0166208. doi: 10.1371/journal.pone.0166208 (PMC5104367; doi:10.1371/journal.pone.0166208)
Supplement: S1 Table — For each subject, table shows treatment order, change in behavioral measures (change from baseline, post-pre), and episode number used in each treatment. (DOCX) [file pone.0166208.s003.docx]

**Table S.1**

| Subject ID | Tx Order | Treatment | ΔMML | ΔVAS | Episode |
| --- | --- | --- | --- | --- | --- |
| 1 | 1 | T | 3 | 0 | D |
| 1 | 2 | C | -1 | 0 | C |
| 1 | 3 | C+T | -3 | -10 | B |
| 1 | 4 | S | 1 | 10 | A |
| 3 | 1 | C | 0 | 2 | B |
| 3 | 2 | T | -3 | -1 | D |
| 3 | 3 | C+T | -1 | -2 | C |
| 3 | 4 | S | 3 | -1 | A |
| 4 | 1 | C | -10 | -15 | D |
| 4 | 2 | T | 4 | -16 | B |
| 4 | 3 | S | -6 | -17 | A |
| 4 | 4 | C+T | -6 | -36 | C |
| 5 | 1 | C+T | -1 | 0 | D |
| 5 | 2 | S | 0 | 0 | A |
| 5 | 3 | T | 1 | 0 | C |
| 5 | 4 | C | -2 | -2 | B |
| 7 | 1 | C+T | -2 | 0 | A |
| 7 | 2 | S | -1 | -5 | B |
| 7 | 3 | T | -6 | -3 | C |
| 7 | 4 | C | 0 | 0 | D |
| 8 | 1 | S | -2 | -14 | B |
| 8 | 2 | C+T | 0 | -4 | A |
| 8 | 3 | C | -5 | -7 | D |
| 8 | 4 | T | 2 | 1 | C |
| 9 | 1 | T | -2 | 0 | D |
| 9 | 2 | C | 0 | -1 | A |
| 9 | 3 | C+T | -5 | -9 | C |
| 9 | 4 | S | 2 | 4 | B |
| 10 | 1 | T | 5 | 2 | A |
| 10 | 2 | C | -2 | -1 | D |
| 10 | 3 | C+T | 2 | 2 | B |
| 10 | 4 | S | 0 | -4 | C |
| 11 | 1 | C | -9 | -14 | A |
| 11 | 2 | T | 1 | -5 | D |
| 11 | 3 | C+T | -5 | -20 | C |
| 11 | 4 | S | -1 | 5 | B |
| 12 | 1 | C | 1 | 3 | C |
| 12 | 2 | T | -1 | -8 | A |
| 12 | 3 | S | 2 | 16 | B |
| 12 | 4 | C+T | 0 | -6 | D |
| 13 | 1 | C+T | 0 | -10 | A |
| 13 | 2 | S | 0 | 0 | C |
| 13 | 3 | T | 0 | -15 | D |
| 13 | 4 | C | 0 | -4 | B |
| 14 | 1 | S | -2 | 0 | C |
| 14 | 2 | C+T | 0 | 0 | A |
| 14 | 3 | T | 1 | -3 | B |
| 14 | 4 | C | 0 | -5 | D |
| 15 | 1 | C+T | 3 | 0 | B |
| 15 | 2 | S | 1 | -5 | C |
| 15 | 3 | C | -1 | 0 | A |
| 15 | 4 | T | 1 | -5 | D |
| 16 | 1 | C | -1 | -4 | B |
| 16 | 2 | T | 0 | 0 | A |
| 16 | 3 | C+T | -2 | 0 | D |
| 16 | 4 | S | 0 | 2 | C |

*C = CAS-only; T = tDCS-only; C+T = CAS+tDCS; S=Sham*
